# Correlations between internal and external ocular factors and macular pigment optical density

**Published:** 2018

**Authors:** Ruxandra Tudosescu, Cristina Mihaela Alexandrescu, Sânziana Luminiţa Istrate, Alexandra Diana Vrapciu, Radu Constantin Ciuluvică, Liliana Voinea

**Affiliations:** * Ophthalmology Department, “Regina Maria” Private Health Care Network, Dorobanţi Hyperclinic, Bucharest, Romania; ** Ophthalmology Department, University Emergency Hospital, Bucharest, Romania; *** Ophthalmology Department, “Carol Davila” University of Medicine and Pharmacy, Bucharest, Romania; **** Division of Anatomy, Faculty of Dental Medicine, “Carol Davila” University of Medicine and Pharmacy, Bucharest, Romania

**Keywords:** macular pigment optical density, blue-light, iris colour, refractive erros, computer using patients

## Abstract

Aim. To assess the relationship between the macular pigment optical density and blue-light issued by computers, glare sensibility, with iris color, age, sex, or refractive errors.

Material and methods. 83 patients (166 eyes) were enrolled in a prospective observational study. They were divided into 2 groups: group 1 (study group) - computer using patients (time spent in front of the computer for minimum 8 hours per day, 5 days per week, 2 years) - 43 patients and group 2 (control group) - 40 patients. The following investigations were conducted in all the selected cases: visual acuity, refraction, biomicroscopy, measurement of the MPOD, glare sensitivity, assessment of eye color.

Results. 51.81% of the patients were included in group 1, while the rest, 48.19%, were in group 2. Thus, the MPOD had a mean value of (+/ -SD) 0.42+/ -0.13 (t = -1.08, p = 0.28) in group 1, and 0.44+/ -0.16 on the LE. The results showed a MPOD mean value of 0.51+/ - 0.16 in group 2 and 0.51+/ -0 .16. (t = 0.49, p = 0 .62) on the LE. 55.77% of the patients with light colored iris and 56.14% of those with dark iris had a low MPOD.

Conclusions. The data from our study failed to illustrate a significant correlation between MPOD and blue-light issued by computers. Furthermore, a statistic significant relationship regarding iris color, refractive errors, glare, and MPOD was not observed.

Abbreviations: L = lutein, Z = zeaxanthin, MZ = meso-zeaxanthin, AMD = age related macular degeneration, MPOD = macular pigment optical density, MP = macular pigment, HFP = Heterochromatic Flicker Photometry, RE = right eye, LE = left eye

## Introduction

The macula lutea is the region of the retina that is responsible for the sharp and high – resolution central vision. The center of the macula contains a small pit – the fovea - that has the highest density of cone photoreceptors, thus providing the best visual acuity. Lutein (L), meso-zeaxanthin (MZ) and zeaxanthin (Z) are the components of the macular pigment. The predominant location of the macular pigment is in the inner plexiform layer and the photoreceptor axon layer of the macula [**1**].

The primary role of these natural xanthophylls is supposed to be the protection against the development of age related macular degeneration (AMD) [**2**]. Among individuals, their concentration and spatial distribution can be very variable [**3**]. Even though the optical density of the macular pigment (MPOD) can have wide ranges, it is very rare to have a MPOD below 0.2 and, in a healthy person, it is not totally absent [**4**,**5**].

Another important role of these carotenoids is the protection against light-induced oxidative stress by the free oxygen radicals, them being antioxidants [**6**]. This way they are involved in the protection of the photoreceptors located in the macula.

The third major role is the improvement of the visual performance (glare, contrast) [**7**].

The concentrations of the MP can depend on various factors such as smoking, exposure to blue light at 499-530 nm, the low intake of L and Z. and obesity, lifestyle, the color of the iris, refractive errors like myopia, low carotenoid levels in the serum and possibly ageing [**8**,**9**-**13**].

Oxidative stress and blue light might be involved in the development of ARM with a possible connection with a low density of the macular pigment [**14**,**15**].

Nowadays, an important source of blue light are the computers and the prevalence of people working with these devices is more and more high. 

Regarding glare, there are recent studies which suggest that a low MP can influence visual performance [**16**]. There are 2 types of glare: discomfort glare – when there is too bright illumination and disability glare - when stray light reduces contrast on the image quality [**7**].

Glare dysfunctions are reported in glaucoma and in several retinal disorders such as AMD or retinitis pigmentosa [**17**-**19**].

Furthermore, photostress recovery, disability glare, and visual discomfort can be influenced by low levels of MP in normal subjects and can be improved by oral supplementation with carotenoids [**16**].

The primary aim of the study was to investigate if there are any relationships between MPOD and computer using patients. 

The secondary aim was to see if there are any correlations with glare and contrast sensibility, with iris color, age, sex or refractive errors. 

## Material and methods

The study was conducted in agreement with the declaration of Helsinki, with an informed consent practice, good clinical and medical practice and the institutional review board regulations. We enrolled a number of 83 patients (166 eyes) in a prospective observational study, conducted from May 2017 to August 2017 in the University Emergency Hospital, Bucharest, Romania – the Ophthalmology Clinic. 

The including criteria for all patients were subjects over 18 years old and under 65 years old, with a minimum BCVA of 0.8. Exclusion criteria for all patients included: corneal diseases that might impair the visual acuity, retinal diseases that might interfere with the measurement of the MPOD, presence of cataract. After providing an informed consent, patients were divided into 2 groups: Group 1 (study group) consisted of 43 patients. Group 2 (control group) consisted of 40 patients. The including criteria for group 1 - the computer using patients was: time spent in front of the computer for a minimum of 8 hours per day, 5 days per week, 2 years. In group 2 - the normal group, we included subjects who did not fit the criteria for group 1.

All the patients in group 1 worked in the informatics industry and the subjects in group 2 worked in the medical field.

The following investigations were conducted in all the selected cases: visual acuity, refraction, biomicroscopy, and measurement of the MPOD, sensitivity to contrast and to glare. We also assessed the color of the eyes, the use of glasses with or without filters against the blue light of the computers. Refraction values were calculated using the spherical equivalent, which represents the sphere’s value plus half the cylinder’s value. 

**Measurement of MPOD**

MPOD was measured using Heterochromatic Flicker Photometry (HFP), with Macular Pigment Screener MPS II (Electron eye technology, Cambridge, United Kingdom) at 0.5° of retinal eccentricity. The MPOD can be calculated through this method by measuring the absorbance of the blue light by the MP. The results of MPOD are values on a scale of 0-1. If the value is low, the level of blue light that reaches the macula is higher.

HFP technique needs patients to match flicker using two wavelengths a blue one at 465 nm absorbed by the MP and a green one, at 530 nm which does not. 

The determination of the minimum flicker point by flicker matches is done by the MPS II in a quick and easy way. All the patients were naive to the testing. They were instructed by a trained examiner regarding the technique and were shown a demonstration. They had to press a button at the detection of the flicker [**20**].

The MPOD values results were divided into 4 groups: 0-0.25 - very low, 0.25-0.5 - low, 0.5-0.75 - good, 0.75-1 - very good. 

**Statistical analysis**

All data was analyzed using SPSS. Using the Shapiro–Wilk test and Kolmogorov-Smirnov test, the data was interpreted for normality. For the normally distributed data, student t test and Pearson correlation coefficient were used for analysis and a p-value < 0.05 was considered as being statistically significant. A descriptive analysis, which included means, medians, and standard deviations, was performed. 

## Results

166 eyes of 83 patients were analyzed in the present study. A number of 63 female patients and 23 male patients were included, 3 times more females than males (female/ male = 60/ 23 = 2.6). 

The mean age for the women was 35.85 +/ -0.99 years, and for the men was a 33.13 +/ -1.93 years.

Regarding optical correction, 63% of the patients did not have any optical corrections. 

Regarding the main type of ametropia, 63.9% of the patients were hypermetropic while the rest of 36.1% were myopic.

The values of the measured MPOD in the right and left eye varied from 0.10 to 0.77, with a means (+/ -SD) of 0.44 +/ - 0.14 in the right eye (RE), and 0.45 +/ -0.16 in the left eye (LE).

39.76% of the subjects had a higher MPOD on the LE, 34.94% had it on the RE, while 25.30% did not have a RE-LE difference. 

**Fig. 1:**
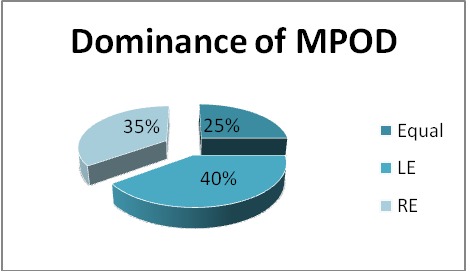
From the statistical data, no significant correlation was found regarding the difference in MPOD between the 2 eyes (p>0.05)

The MPOD difference between the 2 eyes was between a minimum of 0.04 and a maximum of 0.34, with a mean of (+/ -SD) 0.08 +/ -.06. 

The mean value of the MPOD of the 2 eyes was between 0.16 and 0.77 in hypermetropic patients, with a mean value (+/ -SD) of 0.44 +/ -0.15, while in myopic patients the mean value was between 0.14 and 0.72, with a mean value (+/ -SD) of 0.45 +/ - 0.13. 

The mean value of the MPOD of the 2 eyes was between 0.14 and 0.62 in male patients, with a mean value (+/ -SD) of 0.42 +/ -0.11, while in female patients, the mean value was between 0.16 and 0.77, with a mean value (+/ -SD) of 0.45 +/ - 0.15. Taking into consideration the parametric distribution of the data, a t test was performed, showing no statistical significant correlation. 

We analyzed glare in patients in both groups. According to the glare test, the score results were divided into poor, average and good. The results are illustrated in **Table 1**.

**Table 1:** There was no significant statistic correlation between the MPOD and glare of each eye (p>0.05)

%patients	poor	average	good
Glare RE	0%	8.43%	91.57%
Glare LE	1.20%	3.61%	95.18%

**Fig. 2:**
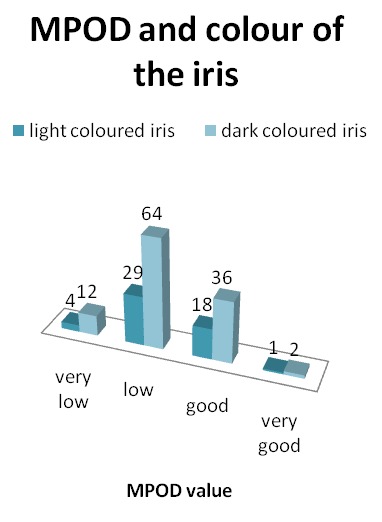
No statistical significant correlation was found between the MPOD value and the color of the iris (p value two- tail 0.72)

Regarding the color of the iris, the subjects were divided into 2 groups, dark colored iris, and light colored iris (green and blue). The results are illustrated in **Fig. 2** and **Table 2**.

**Table 2:** 55.77% of the patients with light colored iris and 56.14% of those with dark iris had a low MPOD

Percentage %	light colored iris	dark colored iris
very low	7.69	10.53
low	55.77	56.14
good	34.62	31.58
very good	1.92	1.75

51.81% of the patients were included in group 1 - computer using group (>8 hours/ day), while the rest, 48.19%, were in group 2 (<8 hours/ day).

The mean age of group 1 was (+/ -SD) of 36.02 +/ - 7.8, and that of those in group 2 (+/ - SD) was de 34.10 +/ - 8.60. 

We analyzed if the time spent in front of the computer influenced the type of ametropia and the results are shown in **Table 3**. 

**Table 3:** There was no statistical correlation between the type of ametropia and the hours spent using the computer when using a Chi-square test

	Refraction	
	hypermetropia	myopia
Group1	26	17
Group2	27	13

We analyzed the MPOD value to see if there was any correlation regarding each eye, the means, and the difference between the 2 eyes. 

Thus, in group 1, the MPOD value of the RE was between 0.10 and 0.67, with a mean of (+/-SD) 0.42 +/ - 0.13 (t = -1.08, p = 0.28) and between 0.10 and 0.77 on the LE, with a mean of (+/ -SD) 0.44 +/ -0.16. In group 2, the results showed a MOPD on the RE, between 0.19 and 0.77, with a mean of (+/ -SD) 0.51 +/ -0.16, and between 0.10 and 0.77 on the LE, with a mean of (+/ -SD) 0.51 +/ -0.16 (t = 0.49, p =0.62).

The difference of MPOD between the 2 eyes was between 0.00 and 0.34 with a mean of (+/ -SD) = 0.06 +/ -0.06 in group 1, and between 0.00 and 0.24 in group 2, with a mean of (+/ - SD) 0.07 +/ - 0.06 (t = -0.61, p = 0.54).

The mean values of the MPOD in group 1 had a range between 0.14 and 0.72, with a mean of (+/ -SD) 0.45 +/ - 0.14, while in group 2, it was between 0.19 and 0.77, with a mean of (+/ -SD) 0.44 +/ - 0.14 (t = 0.41, p = 0.68).

The statistical analysis showed no significant correlation. Therefore, our study revealed that there was no influence of the blue light issued by the computer on the MPOD. 

## Discussions

The results that interest MP determinants are very variable and still inconclusive [**21**]. It seems that there is no consistency between different studies and the results cannot be replicated. The causes can be multiple from sampling to insufficient subjects or measurement issues that prevent us from achieving a significant statistical correlation. Moreover, the MPOD can vary in different geographical areas. 

In our study, we tried to see if we could find some significant correlations regarding various determinants that can influence the MPOD in the Romanian population.

We would like to note that the measurements in our study were done using a HFP method, the most common one used for the measurement of the MPOD. 

Various studies have tried to see if the iris color can influence the MPOD. In their report, Bone and Sparrock (1971) [**22**] showed no significant association between MPOD and the color of the iris, but later on, in their study, Hammond et al. (1996) showed that light colored irises (blue and green) had less MP density than dark colored ones [**8**,**23**]. In our study, the results showed no statistical difference between these 2 types of eye color.

In consistency with current literature [**24**-**26**], our study did not reveal any significant correlation between MOPD and the type of ametropia. Myopic patients did not show a MP density smaller than the hypermetropic ones.

Previous studies tried to see if ocular dominance can influence MP density [**27**]. Even though it is known that ocular dominance can influence visual performances, there is no proven data that can demonstrate a significant relationship between MP density and ocular dominance [**24**]. The results from our study were consistent with these results, showing that there is no difference in MPOD between the 2 eyes. Furthermore, our data showed that the difference in MPOD between the 2 eyes is not statistically significant between males and females. 

To our knowledge, no other study has tried to find differences in MPOD in patients who used computers for a long period of time, these devices being one of the most important sources of blue–light. So, we hypothesized that there could be a low MPOD in normal subjects who have spent more than 8 hours/ day for at least 2 years exposed to computers. However, our statistical data comparing them with normal subjects who did not meet the group 1 criteria, showed no significant statistical difference between the 2 groups. Yet, there are limitations to our study such as small sampling. 

## Conclusions

The role of the macular pigment is to safeguard the photoreceptors from the oxidative stress, improving the visual performances and possibly providing a long-term protection from the development of AMD. The MPOD can be influenced by various determinants like the intake of L and Z, gender, age, obesity, iris color and others, although there are inconsistencies in studies results. 

In conclusion, the data from our study failed to illustrate a significant correlation between MPOD and blue-light issued by computers. Furthermore, no statistically significant relationship was observed regarding iris color, refractive errors, glare, and MPOD.

Although these are negative findings, they are important in further studies regarding the distribution of MP and the effects of blue- light on MPOD. 

**Acknowledgements**

1. AMD Nobel Pharmaceutical is gratefully acknowledged for all the support offered in data collection.

2. All the authors have equally contributed to this study.

**Disclosures**

None
